# Development of a Voice-Activated Virtual Assistant to Improve Insomnia Among Young Adult Cancer Survivors: Mixed Methods Feasibility and Acceptability Study

**DOI:** 10.2196/64869

**Published:** 2025-03-10

**Authors:** Hunter Groninger, Hannah Arem, Lylian Ayangma, Lisa Gong, Eric Zhou, Daniel Greenberg

**Affiliations:** 1 MedStar Health Research Institute Washington, DC United States; 2 MedStar Washington Hospital Center Washington, DC United States; 3 Georgetown University School of Medicine Washington, DC United States; 4 MedStar Health Research Institute Hyattsville, MD United States; 5 Dana Farber Cancer Institute Boston, MA United States; 6 MediaRez LLC Washington, DC United States

**Keywords:** cancer, survivor, insomnia, cognitive behavioral therapy, technology, app, oncology, mobile health, artificial intelligence, young adults, sleep, mHealth, mobile health, CBT, voice-activated virtual assistant, virtual assistants, focus group, qualitative research

## Abstract

**Background:**

Up to 75% of young adult cancer survivors (YACS) experience chronic insomnia, negatively affecting physical and emotional health and overall quality of life. Cognitive behavioral therapy for insomnia (CBT-I) is a gold-standard intervention to address insomnia. To improve CBT-I access and treatment adherence, screen-based digital CBT-I platforms have been developed. However, even with these digital products, widespread uptake of CBT-I remains limited, and new strategies for CBT-I delivery are warranted.

**Objective:**

The objective of this study is to understand how YACS experience insomnia and how they might incorporate technology-delivered CBT-I into a daily routine and test the feasibility and acceptability of a novel screen-free voice-activated virtual assistant–delivered CBT-I prototype.

**Methods:**

Eligible participants—ages 18-39, living with a history of cancer (any type, any stage), self-reporting on average less sleep than National Sleep Foundation recommendations, and English-speaking—were recruited from a major urban cancer center, 2 regional oncology clinics, and 2 cancer survivorship support groups. We conducted 4 focus groups to understand the YACS experience of insomnia, their routine use of technology at home, particularly voice-activated virtual assistants such as Amazon Alexa, and input on how CBT-I might be delivered at home through a smart speaker system. We developed a prototype device to deliver key elements of CBT-I at home along with circadian lighting and monitoring of post-bedtime device use, collected YACS user perspectives on this prototype, and then conducted a single-arm feasibility and acceptability study.

**Results:**

In total, 26 YACS (6-7 participants per group) experiencing insomnia participated in focus groups to share experiences of insomnia during cancer survivorship and to provide input regarding a CBT-I prototype. Common triggers of insomnia included worry about disease management and progression, disease-related pain and other symptoms, choices regarding personal device use, and worry about the impact of poor sleep on daily functioning. In total, 12 participants completed device prototype testing, engaging with the prototype 94% of the assigned times (twice daily for 14 days; meeting predetermined feasibility cutoff of engagement ≥70% of assigned times) and rating the prototype with an overall mean score of 5.43 on the Satisfaction subscale of the Usability, Satisfaction, and Ease of Use scale (range 4.42-7; exceeding the predetermined cutoff score for acceptability of 5.0). All participants completing the study reported they would be interested in using the prototype again and would recommend it to someone else with insomnia.

**Conclusions:**

YACS were highly engaged with our voice-activated virtual assistant–delivered CBT-I prototype and found it acceptable to use. Following final device development, future studies should evaluate the efficacy of this intervention among YACS.

**Trial Registration:**

ClinicalTrials.gov NCT05875129; https://clinicaltrials.gov/study/NCT05875129

## Introduction

Insomnia, characterized by the struggle to initiate or maintain sleep or the experience of nonrestorative sleep, brings along symptoms that hinder daily functioning, such as weariness, concentration difficulties, and mood disruptions [1]. Among the estimated 650,000 young adult cancer survivors (YACS, ages 18-39 years old) in the United States, up to 75% are grappling with insomnia symptoms, with nearly 30% likely to experience insomnia disorder [2-8]. Untreated insomnia is associated with a myriad of psychological and medical consequences, exacerbating the long-term impact of cancer diagnosis and treatment [3,4,9-11].

Insomnia adversely affects cardiometabolic and immune system health, neurobehavioral function, depression, fatigue, and overall quality of life [9,12-14]. These repercussions can also result in reduced physical activity and increased sedentary behavior, both linked to negative health outcomes in YACS [4,15]. In comparison to those with regular sleep patterns, cancer survivors dealing with insomnia tend to have more medical visits, double the hospitalization rate, and a higher reliance on medication [16]. Furthermore, insomnia serves as a risk factor for various mental illnesses, with the greatest risk ratios observed in depressive disorders. Despite its persistent nature during and after cancer treatment, discussions about insomnia with health care providers remain infrequent [17]. Addressing the need for accessible and effective insomnia treatment targeted to support this population with distinct cancer trajectories due to diagnosis at a young age is imperative.

Cognitive behavioral therapy for insomnia (CBT-I) is the gold standard intervention recommended by the National Comprehensive Cancer Network (NCCN) to address insomnia in cancer survivor populations [18-20]. Previous studies have demonstrated the effectiveness of CBT-I among YACSs [21,22]. However, widespread accessibility to CBT-I remains challenging due to an insufficient number of trained providers, a lack of insurance coverage, and even language barriers [23,24]. The administration of CBT-I demands specialized training, and its delivery, typically over 5-6 in-person sessions, can be time-consuming and expensive, creating multiple obstacles to care. Though CBT-I remains the gold standard intervention, adherence can be challenging due to a variety of patient and treatment-related factors as well as multiple changes to bedtime required by the CBT-I component called sleep restriction [25,26]. Even with shorter treatment plans of 3-4 sessions, dropout rates in trials have reached 20%-30%, suggesting potential challenges in real-world adherence [27,28].

In response to this, internet-based or app-delivered digital automated therapies have been developed and proven easier to access by patients, though many of these programs are more instructional than interactive, which may be less engaging for participants [29-31]. Such platforms can make therapy more flexible and accessible, particularly for individuals without access to in-person therapy or who prefer to engage with CBT-I at home. Several meta-analyses and reviews indicate that electronic delivery of CBT-I is just as effective as traditional, face-to-face approaches [29,30]. These programs are grounded in evidence-based practices and typically incorporate components like those found in traditional CBT-I [32,33]. However, there are concerns that the self-directed format of these programs might lead to lower treatment adherence [34]. Some critics also point to a lack of comprehensive quality control over these digital therapeutics as a potential issue [34,35]. Furthermore, these internet-delivered therapies require user engagement with digital screens right at bedtime, potentially confounding the physiologic process of melatonin secretion that enhances sleep [36].

Building on prior work [37], we hypothesized that we could address these issues—improving access to CBT-I, greater technology usability, and reducing screen-time use—at once by integrating 3 distinct technologies running custom software and that YACS would find this technology appealing to use. First, we aimed to deliver the most important therapeutic components of CBT-I interactively via an AI-assisted smart speaker system and without the use of mentally and emotionally activating, melatonin-suppressing screens at night. Second, we deployed smart lighting controls to facilitate CBT-I suggested changes to the room environment. Finally, we used a customized router that provided feedback to each participant about post-bedtime screen use. We supported the ability of YACS to make different choices about screen use at night. We believe these 3 technologies could work together in a customized package to meet the best elements of conversation CBT-I with new ways to support adherence through complex and lengthy treatment protocols. Thus, using an approach involving active stakeholder input and an iterative design process, we aimed to develop and test a novel prototype to increase sleep duration for YACS.

## Methods

### Participants and Recruitment

Recruitment occurred via patient referrals from 11 oncologists at a large urban academic medical center in Washington, DC, from 2 regional oncology practices focused on YACS populations, 2 regional cancer survivor support groups, and Facebook social media advertising. The Washington, DC-based academic medical center annually provides oncology care to over 2000 adult patients living with cancer, including approximately 360 YACS. Regional young adult oncology practices included a large outpatient clinic in Northern Virginia and a similarly large clinic in Charlotte, NC. Regional cancer survivor support groups were based in Washington, DC, and suburban Maryland in the Baltimore-Washington, DC region. In all settings, approved recruitment flyers were placed strategically in clinic waiting areas or were shared directly with potential participants by providers (oncology clinics) or support staff (cancer survivor support groups). Members of the study team were available by phone or email to answer questions. Potential participants were screened for eligibility by the research team.

Participants were included if between 18 and 39 years old, living with a history of cancer (any type, any stage), self-reported to average less sleep than recommended for their age by the National Sleep Foundation for ≥3 months (7-9 hours per night) [1], could engage with the study prototype and complete surveys and interviews in English, and had Wi-Fi access at home. Individuals were excluded if they were enrolled in hospice care or lived outside the continental United States.

### Focus Groups

Our research team followed the consolidated criteria for reporting qualitative research (COREQ) process through this qualitative study [38]. Qualitative data was gathered through 4 formative focus groups led by study authors (HG and DG), held virtually between December 2022 and March 2023. During recruitment, potential participants were informed that the team sought to learn more about the experience of chronic insomnia and gather perspectives on the use of mobile technology to improve sleep at home. Prior to the study, no study team member had any relationship with any participant. We included 26 participants (6 in focus group 1; 7 in group 2; 7 in group 3; and 6 in group 4). Prior to joining the virtual meeting, participants were asked to turn off their video cameras and deidentify their names (eg, “Guest” or “Participant”) making participation anonymous. Focus group questions and prompts—outlined in Table 1—were developed by members of the research team based on prior work with cancer survivors and tested with 2 YACS who were not part of the focus groups. Each focus group consisted of a brief introduction to the project followed by a facilitated conversation regarding participant experiences of insomnia and sleep disruption in cancer survivorship. Our inquiries explored participant insomnia experiences, elucidating perceived causes and symptoms. Participants were also asked about the current use of technology at home, particularly smart technology such as smart speakers and other voice-activated virtual assistants, screen use habits, and concerns about privacy related to smart technology use. Each focus group lasted approximately 90 minutes. Members of the research team continually reviewed focus group content and, after the fourth focus group, determined that content saturation had been achieved.

**Table 1:** Focus group questions aimed at understanding chronic insomnia of young adult cancer survivors and portable technology habits.

Theme	Questions
Perceptions and experiences of insomnia	What is your insomnia experience like? How long has it been occurring? How has insomnia affected your cancer journey?
Mitigation strategies for insomnia	What steps have you taken to improve your sleep? What have you found to be helpful, if anything?
Device use and habits	Which connected devices do you use every day?
Associated between device use and sleep habits	To what extent do you think that the use of connected devices affects your sleep?
	Have you taken any efforts to try and change your connected device use, specifically as it relates to how you sleep? If yes, what have you found to be helpful? If not, what would interest you to make changes?
VAVA^a^ technology use and habits	Do you use VAVAs at home or elsewhere? Examples include Apple Siri, Amazon Echo/Dot, or Google Assistant. If you have not used VAVAs, why not?
Comfort using VAVA-delivered strategies to improve insomnia	What are your thoughts on a smart speaker system giving you instructions like this about your sleep? What other ways do you think that technology might be able to help make better choices to improve your sleep?
Familiarity with smart lighting and integration into sleep-wake experience	What is your familiarity with smart lighting? What are your thoughts on having smart lights installed in your bedroom that would automatically change in the evening to set up an optimal bedtime for you? What if the smart lights also automatically changed prior to an optimal wake time?
Screen time usage and device prototype feedback about habits	What are your thoughts on seeing weekly or monthly reports on how often your devices impact your sleep, and which devices have the most significant effect on your sleep? Would you have privacy concerns about a system that provides feedback about your internet usage to you? If yes, how do these privacy concerns compare to your privacy concerns about other apps you use and websites you visit? Would those privacy concerns change your interest in using a system like this for better sleep? What system would make you feel confident about privacy protection on a system like this?

^a^VAVA: voice-activated virtual assistant.

### Device Prototype Development

**Figure 1:**
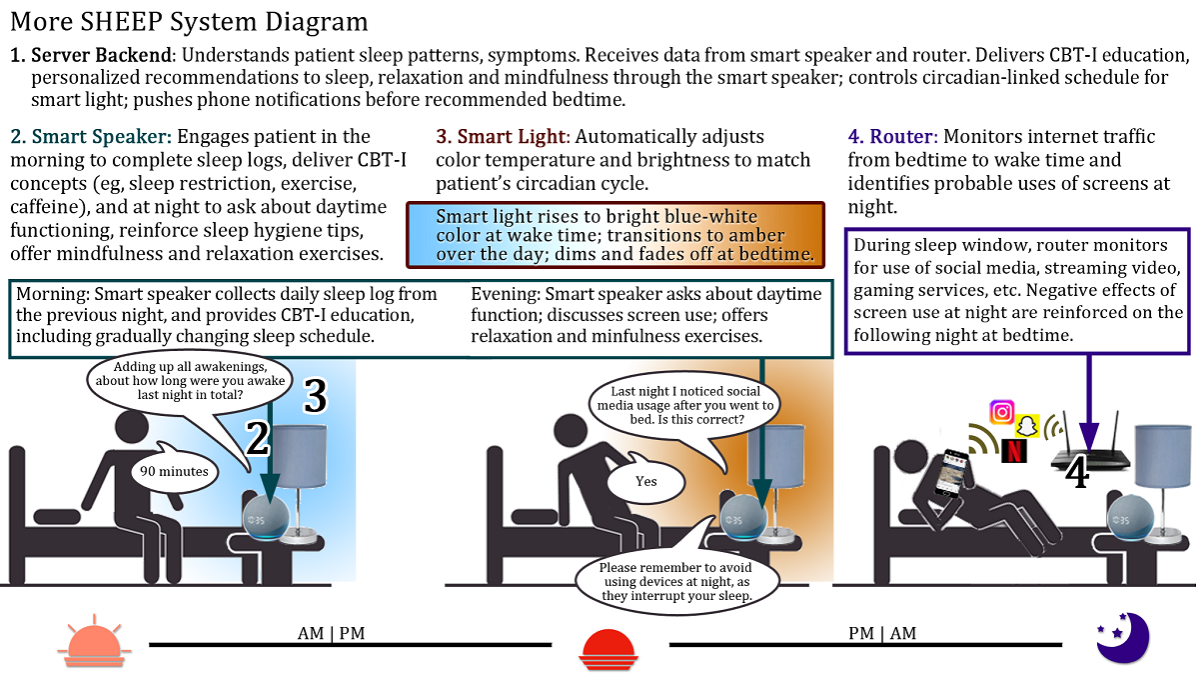
The More SHEEP (More Sleep Hours Electronic Education Program) system connects a smart speaker, smart lighting, and unique internet router to provide tailored cognitive behavioral therapy for insomnia (CBT-I) and individualized screen use data to individuals with chronic insomnia.

The More SHEEP (More Sleep Hours Electronic Education Program) system includes proprietary Media Rez technology for delivering elements of CBT-I via natural voice interactions for more engaging patient education and patients can complete sleep logs without bedtime screen use (Figure 1). The team used the first 2 focus groups with YACS to design the systems and 2 follow-up focus groups to iteratively revise the software. The system was built around an AI-driven smart speaker using an Amazon Alexa device (Echo Dot 3rd Gen). In preparation for the 2-week trial, the technical team reduced the 6-session course of CBT-I to only 2 weeks by concentrating material from later weeks into the earlier sessions, with the goal of providing a comprehensive experience of virtual CBT-I sessions in only 2 weeks. The team also provided a lamp with a color-changing Philips smart bulb controlled by our custom software, which changed its on and off times based on each patient’s unique sleep restriction and sleep consolidation recommendations according to the rules of CBT-I. As a result, the smart lamp bulbs automatically changed color temperature throughout the day. It began with a bright white with blue wavelengths in the morning to suppress melatonin production and reduce sleepiness and shifted to a warm amber in the evenings, so melatonin is not suppressed, and the patient can feel naturally sleepy [39,40]. The technical team also developed router control software to record and categorize the web-based traffic (social media, streaming video, and gaming) by the participant after bedtime (but not other members of the household, by having only the participant use the WiFi from the included custom router), so this screen use information could be presented to each patient to help them make better decisions about evening device use. For example, the system compares IP addresses visited by the patient with known sites like Netflix, Call of Duty, and Instagram. The system was also built to intelligently filter out other sources of internet use, like automatic updates and other forms of device telemetry. In this system, patient privacy and confidentiality are ensured since the system immediately discards all identifiable information about post-bedtime website usage once that usage is shared with the patient. No information is collected about websites visited at any other time. Because this IP information is not stored, it cannot be summarized to participants or accessed at a future time, either by the participant or by a third party such as an internet hacker. No information is collected from other people in the household who are using conventional WiFi and not using this system. Together, these 3 elements combine to deliver a comprehensive, innovative sleep solution.

### Device Testing

Following prototype development and refinement, we conducted a single-arm study to evaluate the feasibility and acceptability of the system. While the research team remained knowledgeable about each participant, the technology company Media Rez was blinded to participants to preserve anonymity. For this phase, we screened 27 potential participants for participation, 6 of whom expressed interest and then did not respond to further communication, 5 changed their minds about participation after hearing more information about the study, 1 person wished to proceed with consent but was about to leave the country for an extended travel period, 1 person decided not to participate due to an upcoming medical procedure, and 1 person was initially eligible and then became ineligible due to a hospice referral. We thus recruited and enrolled 15 eligible YACS who were not involved in the focus groups.

After completing written consent, participants received (either by mail or picked up in person) a study materials box containing the prototype smart speaker, smart lamp, router, and setup instructions. A member of the study team assisted with device setup via a brief phone call with each participant. Participants were instructed to engage with the prototype system twice daily for 14 days, including entering sleep logs, receiving smart speaker–delivered education about relevant content (eg, sleep hygiene, relaxation strategies), and reviewing personalized data regarding screen time use. If challenges occurred during system setup or use during the 14-day study period, a research team member contacted the tech team directly for troubleshooting advice to preserve the anonymity of the participants to the tech team.

Study feasibility was determined by user engagement with the prototype as assessed by: (1) the number of days over the study period that participants interacted with the system (≥70% interaction days used as cutoff) and (2) the average number of daily interactions with the prototype (≥2 daily interactions used as cutoff). Study acceptability was assessed by an average score ≥5 (“somewhat agree”) on the Satisfaction subscale of the Usability, Satisfaction, and Ease of Use scale; this subscale is composed of 7 items measuring satisfaction with program content and the method of program delivery [41]. Additionally, all participants completed a semi-structured interview to assess overall user experience, with a focus on overall impressions of the system as well as each specific component, enthusiasm for longer-term use, and recommendations for device improvements.

We used a grounded theory approach for qualitative data analysis. Focus groups (formative phase) and semi-structured interviews (following device testing) were audio recorded and transcribed. Coding was led by HG (trained in qualitative analysis) with assistance from other team members (LA, LG) and reviewed by members of the research team. Initial coding of qualitative data was descriptive in nature, based on the interview guide structure. From the focus group data, emerging themes and exemplary quotes were extracted to develop the prototype prior to testing. Semi-structured interview data will be used to further develop the device prior to future efficacy testing. All quantitative data (following device testing) were summarized using descriptive statistics.

### Ethical Considerations

Per institutional guidelines at the primary study site, this study was first reviewed and approved by the Georgetown University Lombardi Comprehensive Cancer Center Scientific Review Committee prior to review and approval by the MedStar Health Research Institute Institutional Review Board (number 00005548). Focus group participants provided verbal consent to participate at the beginning of each focus group conducted. Participants for device feasibility testing provided written consent prior to the device prototype being shipped to the home. Per protocol approved by the IRB, all identifiable participant data was stored on password-protected, encrypted servers. Focus group participants each received a $30 gift card. Device testing participants were offered a choice to either (1) keep the study device after a factory reset or (2) receive a US $50 gift card.

## Results

### Focus Groups

In total, 26 YACS experiencing insomnia participated in focus groups to share experiences of insomnia during cancer survivorship and to assess the practicality of using this system for delivering components of CBT-I (Table 1). All 4 focus groups echoed similar experiences with insomnia, describing cancer survivorship as characterized by insufficient sleep and diminished energy, whether struggling to initiate sleep or stay asleep, and often finding themselves awake in the middle of the night. Common threads in perceived triggers and symptoms encompassed anxiety and worry about disease management and progression, disease-related pain and other symptoms, choices regarding personal device use, and worry about the impact of poor sleep on daily functioning (Table 2).

**Table 2:** Sample scripts for MORE Sheep modules

Module name	CBT-I component
**Education**	
*Another thing I will do is automatically adjust the provided smart lamp. Tonight, beginning an hour before your bedtime of 10:00 P.M., the lamp will begin to fade out, and turn itself off just before bed. In the morning, it will turn on at its lowest brightness and slowly get brighter until it is fully bright at your wake-up time of 7:00 A.M. As I learn more about your sleep patterns, I may suggest changing when you sleep, and the lamp will act as a visual cue of your sleep schedule. In the coming days, I'll explain the effects of light, your circadian rhythm, and how your body uses a hormone called melatonin to regulate sleep.*	Psychoeducation
*Congratulations! You left the bed four times this week when you couldn't sleep and listened to three relaxation scripts until you were tired enough to go back to the bedroom to sleep. This is good, because it helps you associate the bed only with sleep, and not with lying awake. In this case, sleep restriction could be helpful, making your total time in bed closer to your actual sleep time. Instead of a 10:00 pm bedtime, would you like to try going to bed at 10:45 for the next few nights?* If Yes: *Good. I'll reset your alarms and lights for 10:45, and we'll see how that works out.*	Psychoeducation and stimulus control
**Morning**	
*We worked together to establish a bedtime of 10:45 and wake time of 7 A.M. The hours I set for your sleep are not a lifelong sentence. It was merely the necessary step to get you to try to improve your sleep. As you may be starting to see, sleep restriction and stimulus control are meant to help consolidate your sleep to improve your ability to fall asleep and stay asleep.*	Sleep restriction
*Rate the quality of your sleep last night from 1-5 (with one being the best and five being the worst). About how many times did you wake up last night?*	Sleep hygiene
**Night**	
*Being active each day is important. Even a 10 minute walk is a great start! Would you like me to remind you to get moving during the day?* If Yes: *Great. What time can I send you a push notification on the app to be active?* User Time Input: *Ok, I'll send you a notification to be active each day at 1:30 P.M.*	Psychoeducation
*Please rate your tiredness today on a scale of 1 to 5, with 1 being the least tired and 5 being the most tired.*	Sleep Hygiene
*Last night I detected internet usage during your sleep window. Specifically, it appears you used devices late at night. This activity appears to include streaming video. Is this correct?* If Yes: *Remember that this will hurt your sleep. Let me give you a tip for tonight: Establish a bedtime routine where you give yourself time to check devices one more time and then commit to keeping them off until morning. It should ideally be 30 minutes or more before bed. Give yourself the time before bed to view social media or watch a show, but promise yourself that once it is bedtime, your devices are off limits. Consider turning on a “do not disturb” mode, or fully power off these devices, and keep them away from the bed.*	Psychoeducation, Sleep Hygiene
**Relaxation**:	
*For this first relaxation exercise, I'd like to play a few samples of each type. In the future, I'll help you find which type you enjoy most.*	Relaxation

I think most times I get [insomnia] it was a little bit combination of stress and having emotional traumas that led to sleeplessness.

What causes me to stay awake is when I am having some level of depression and anxiety.

At the end of the day, [if] you sleep one or two hours and wake up, the experience is bad. A lot of people are looking for how you can get yourself calm. There is a lot of anxiety and depression [living with cancer].

Use of personal technologies was frequently cited as a trigger for insomnia:

Sometimes these [screen] devices become a problem for me in terms of sleeping. I put them in Airplane mode so that I can get better sleep… [but if] I forget to put it on Airplane mode and if my phone disturbs me, I find it difficult to fall asleep again.

In order not to get distracted, sometimes [I find myself] setting a limited time to spend with my device and try to adhere to it. And when it is time [to stop phone use] I know it's time to quit time to go to bed, though it is really not easy.

Common strategies to mitigate sleep disturbances included web- or app-based meditation programs to calm anxiety, worry, or distraction through screen devices. Participants frequently noted a reluctance to seek pharmacotherapies for insomnia, voicing a preference for nonpharmacologic interventions.

I try to use [apps to] wind down stuff on my phone. I try to make sure that [I] turn off any screen usage and make sure that I am on my bed getting ready to sleep.

I think about a smart speaker for therapy, it is something very unique. It [makes] you feel like [you are in] a relationship, like talking to someone you know. So, for me personally I think it is going to help positively to help [instead of] the stress of going to see therapist from day to day, time to time, consider appointments, traffic, and out of pockets, financially.

You know when you have such a device around you, it can literally put you back to sleep. You do not need pills.

Participants were familiar with smart speakers or voice-activated devices. All participants reported using smartphone-based voice functions, like Apple Siri, for straightforward tasks (eg, creating a calendar reminder) or information gathering (eg, local weather). A minority used in-home smart speakers, primarily to play music or deliver news. All participants expressed interest in the concept of AI-delivered CBT-I and agreed that a smart speaker delivery system could promote frequent engagement with this therapy in the convenience of home. One participant was familiar with smart lighting but did not use it regularly. The remaining participants expressed interest in incorporating smart lighting into a home-based insomnia program.

You know when you are in a room where the lighting systems feels comfortable for sleep, you realize you can sleep more better and easier than compared to when we are in a room whereby you get you know more blue light. So that tends to be a bit of a problem to sleep and also affects the entire sleeping pattern.

My thought here is that having a smart lighting in the bedroom is what will improve our sleep, our sleeping pattern. Because a lot of people have some type of insomnia because of depression, anxiety, or one thing or the other. But I believe with the installation of smart lighting, the color changes, and the variation of the lighting system, patients might be forced to sleep at a particular time.

Regarding the additional internet router function to monitor and provide feedback about personal device use, all agreed such data could promote making different choices about screen time use. A minority of participants expressed some concern about privacy with router monitoring of internet usage but also expressed acceptance with additional information about prototype privacy protection.

Anything that has to do with privacy, you have to be very careful because you don’t want [personal information] to go out there and fall into the wrong hands.

At least every device connected to a router must have its own IP address. So, I don’t know because each of the IP addresses must have an IP address which can be used to access each of the devices that is connected to a router. I don’t know how [you are] going to secure that in terms of privacy of information or data.

No participants expressed worry about privacy due to the smart speaker itself. A few participants appreciated the possibility of being more open with AI technology than with an actual in-person therapist.

It is like meeting a therapist, it's more like talking to a human, it actually saves us the stress of having to access any health care and I'm talking about having discussion about insomnia or sleep disorder. And it is safe for me having it around me in my [home] environment…goes a long way to have a kind of communication level that will ease the tension of having to say whatever you are feeling.

The most interesting part of it to me is that the therapist’s [voice] in the device can make you feel comfortable when you are trying to make sure you get good sleep.

### Device Testing

In total, 15 participants had an average age of 30.6 (SD 6.8); 10 identified as women; 8 identified as Caucasian, 7 identified as Black or African American, and 1 identified as Hispanic. Of 15 enrolled participants, 2 became lost to follow-up immediately after enrolling (sent the device prototype but never initiated the program), and 1 completed 1 day of participation and then dropped out from concerns about using home Wi-Fi for the project as they were also using high-security home Wi-Fi features for work. The remaining 12 participants completed the study protocol, including a survey and a semi-structured interview.

Participants were able to adhere to the More SHEEP program directions, engaging with the prototype 94% of the assigned times (twice daily for 14 days) and meeting the predetermined feasibility cutoff of engagement ≥70% of assigned times. Participants also found the More SHEEP prototype acceptable, with an overall mean score of 5.43 on the Satisfaction subscale of the Usability, Satisfaction, and Ease of Use scale (range 4.42-7), exceeding the predetermined cutoff score for acceptability of 5.0. All participants completing the program reported they would be interested in using the prototype again, would recommend it to someone else with insomnia, and would be interested in participating in future studies evaluating new iterations of the prototype system.

Following participation, interview themes informed individual experiences with each of the More SHEEP system components (smart speaker, smart lighting, router, and More SHEEP smartphone app) (Table 3).

**Table 3:** The authors enrolled 15 young adult cancer survivors to participate in a single-arm feasibility-acceptability study of the More SHEEP prototype that included qualitative data collection to inform future prototype development. Participant-generated themes inform prototype feasibility, acceptability, and areas for potential improvement.

Prompt	Content
Familiarity with smart device use	*I have another [smart speaker] device and my grandfather has one too because he’s blind. This one definitely had a more pleasant voice (Participant #1)*
Feasibility of use	*The app reminded me twice a day on my phone and it was easy to spend a few minutes with the program before bed or after getting up in the morning. No problem at all (#3)* *For the most part I found [the smart speaker] easy other than when I asked for something that it didn’t know how to give me. It understood what I was talking about and was easy to use (#12)*
Acceptability of use	*I thought it was all pretty easy to use and get used to. I even tried taking it on a visit to family and it worked there for me too. (#6)* *I do the check-ins each morning and evening. I think I remember to do it almost every day, so it’s pretty easy to do, particularly with it being just audio, rather than having to go to a website and read [material]*
Challenges with smart lamp	*I ended up not using the lamp at all because my partner goes to bed at different times so it was disruptive. The rest was easy to use (#4)* *I did sometimes modify my answers to make the smart lamp go off a little earlier…I would find myself throwing a blanket over the top (#1)*
Relevance of CBT-I^a^ language and setting	*Occasionally, the [software] voice mentioned something related to cancer survivorship and I really felt that this program was designed for someone like me (#2)* *I think the awareness [of my sleep choices] was one thing I wasn’t expecting to just pick up on…being more aware of how I felt [around bedtime] (#1)* *I feel like it had surprisingly little survivorship information. For the most part, I felt like I was learning general sleep habits (#12)* *I did think that this [home system] is very convenient as somebody who has three small kids and cancer and a husband so I don’t have to go anywhere and I feel like I’m getting something from these sessions (#11)*
Preference for more customization	*It would be really cool if there was a way to make the lamp different colors maybe depending on the education happening or on my mood. Maybe combine the speaker and the lamp into one, that would be good too (#7)* *I like being able to use sounds at night to sleep. I put the thunderstorm [track] on, or the ocean, and I’m able to sleep (#14)* *It would definitely be nice if [the system] could say, if you could ask how much time you have or something like that*
Need to design for varying spaces or needs	*I’m in college and have roommates so I wasn’t sure if I should try this at school or at home. I’m participating from home but if I used this at school it would have to be different (#10)* *I have a 4-year-old and she can't sleep by herself so she's coming into my room; then I think, can I activate Alexa without waking her up?*

^a^CBT-I: cognitive behavioral therapy for insomnia.

## Discussion

### Principal Findings

We discovered a keen interest among participants in this technology in this preliminary investigation into the experiences of YACS dealing with insomnia and smart home devices, particularly when tailored for user-friendly personalization. Most participants expressed significant interest in using this technology. Participants generally reported an enhanced understanding of CBT-I and its potential impact on mitigating chronic sleep disruption and rated the program as both feasible and acceptable. Participants highlighted the high feasibility of daily sleep logs during our demonstration, a crucial component of successful engagement with CBT-I. This observation implies that the data gathered by the smart home device can be leveraged by AI programming to generate personalized recommendations and schedules, surpassing the scope of conventional sleep hygiene education.

Feedback from participants also informed areas for prototype improvement. Some participants felt the More SHEEP content could speak more to the YACS experience of insomnia and could include more information and recommendations directed specifically to cancer survivorship; in collaboration with young adult cancer survivorship experts, future device iterations could include more language targeted to YACS. Participants often noted that a smart speaker or smart lighting may not be conducive to sharing sleep space. For example, the smart light might remain on to address sleep restrictions per CBT-I protocol for a participant while a bed partner wants to sleep without lighting. Similarly, a participant might not want to awaken others using a smart speaker at bedtime—this was particularly important to 1 participant living in a university dormitory. Finally, some participants expressed preferences for more sleep-related content, like sleep music or white noise, allowing more customizability. Formative testing processes of future prototype iterations may address these concerns, ultimately enhancing participant engagement and treatment adherence.

Developments in home technology play an increasing role in improving insomnia and related symptoms by providing tools and solutions to address sleep disturbances. Examples include but are not limited to mobile sleep-tracking devices, light therapy, smart thermostats, biometric feedback devices, and relaxation or meditation apps [42-46]. As a gold standard for the management of chronic insomnia, implementation of CBT-I continues to be explored in this space, primarily through mobile device applications [47-51]. Nevertheless, even through the use of mobile technologies, maximizing treatment adherence remains challenging. In total, 1 study specifically focused on YACS, delivering an internet-based CBT-I intervention over 6-9 weeks and finding that, while outcomes of insomnia, daytime sleepiness, fatigue, and quality of life improved at the study’s 2 timepoints, most participants did not complete the assigned protocol [52]. Compared to this and other device-driven insomnia mitigation strategies, our prototype is unique in its CBT-I delivery through a smart speaker, limiting device screen exposure whose blue wavelength light can impede nighttime melatonin secretion, as well as smart lighting use that can provide important diurnal visual cues (and block blue wavelength light at night). While most of our study participants had never used these technologies before, acceptability was high, and participants were able to easily engage with the program structure. While we believe that our prototype’s inherent ease of use through voice activation in the bedroom will improve long-term CBT-I treatment adherence, we did not design this early study to evaluate this.

Several limitations of this project deserve mention. First, our insights come from a small number of participants, primarily due to the constraints of the study's limited timeframe. Nevertheless, this sample size appropriately aligns with stage I (intervention refinement) of the NIH stage model for behavioral intervention development. Additionally, as we reached the conclusion of data collection, the absence of emerging additional themes in our qualitative data suggested a saturation of feedback. Second, we note selection bias, as some eligible YACS learned of the study but chose not to participate. We also were not able to complete data collection for 2 enrolled participants. Furthermore, although some information was revealed during post-participation interviews, we did not collect data regarding other factors that might influence the choice to enroll or study adherence, such as income, living situation, or education that might influence outcomes. Given the nature of the intervention, it is possible these same factors could also influence clinical outcomes related to insomnia—thus, future efficacy studies of our prototype should, at a minimum, collect such patient-level data and potentially ensure diversity of participants through purposive recruiting strategies, increasing generalizability of results. By design, this study intentionally did not evaluate clinical outcomes such as improvement in insomnia, mood, daytime fatigue, etc, focusing specifically on device feasibility of use and acceptability over 14 days. While this time frame facilitated participant experience with the More SHEEP prototype, a full CBT-I treatment typically requires approximately 6 weeks; therefore, future prototype refinement may benefit from piloting a longer period of use at home. Nonetheless, this early development phase focused on evaluating device feasibility and acceptability and we believe we have achieved our primary goal. Subsequent studies will delve deeper, refining and testing the prototype for practical feasibility and effectiveness.

### Conclusions

This study combined end-user participant input toward the development of a novel smart speaker, smart lighting, or a router system to deliver key elements of CBT-I for YACS to be used in the home setting. Now that the system has demonstrated feasibility and met acceptability benchmarks, the prototype can continue to be developed to incorporate suggestions from our participants and subsequently be tested for efficacy in the YACS population. Ultimately, this system has the potential to increase much-needed access to CBT-I among cancer survivors.

## Abbreviations

CBT-I: cognitive behavioral therapy for insomnia
More SHEEP: More Sleep Hours Electronic Education Program
YACS: young adult cancer survivor

## Data Availability

The datasets generated during or analyzed during this study are available from the corresponding author on reasonable request.
